# Total testosterone is not associated with lean mass or handgrip strength in pre-menopausal females

**DOI:** 10.1038/s41598-021-89232-1

**Published:** 2021-05-13

**Authors:** Sarah E. Alexander, Gavin Abbott, Brad Aisbett, Glenn D. Wadley, Jill A. Hnatiuk, Séverine Lamon

**Affiliations:** Institute for Physical Activity and Nutrition (IPAN), School of Exercise and Nutrition Sciences, Deakin University, Geelong, VIC Australia

**Keywords:** Physiology, Endocrinology

## Abstract

The aim of this study was to examine the relationship between endogenous testosterone concentrations and lean mass and handgrip strength in healthy, pre-menopausal females. Testosterone has been positively associated with lean mass and strength in young and older males. Whether this relationship exists in pre-menopausal females is unknown. Secondary data from the 2013–2014 National Health and Nutrition Examination Survey were used to test this relationship. Females were aged 18–40 (n = 716, age 30 ± 6 years, mean ± SD) and pre-menopausal. Multivariate linear regression models were used to examine associations between total testosterone, lean mass index (LMI) and handgrip strength. Mean ± SD testosterone concentration was 1.0 ± 0.6 nmol L^−1^ and mean free androgen index (FAI) was 0.02 ± 0.02. In pre-menopausal females, testosterone was not associated with LMI (β = 0.05; 95%CI − 0.04, 0.15; *p* = 0.237) or handgrip strength (β = 0.01; 95%CI − 0.11, 0.12; *p* = 0.926) in a statistically significant manner. Conversely, FAI was associated with LMI (β = − 0.03; 95%CI − 0.05, − 0.02; *p* = 0.000) in a quadratic manner, meaning LMI increases with increasing FAI levels. Handgrip strength was not associated with FAI (β = 0.06; 95%CI − 0.02, 0.15; *p* = 0.137)*.* These findings indicate that FAI, but not total testosterone, is associated with LMI in pre-menopausal females. Neither FAI nor total testosterone are associated with handgrip strength in pre-menopausal females when testosterone concentrations are not altered pharmacologically.

## Introduction

The maintenance of skeletal muscle mass and function is essential for health and quality of life across the lifespan. Peak muscle mass and strength in mid-life are significant indicators of the development of sarcopenia in later life^1^. Gaining a fundamental understanding of the determinants underlying the regulation of muscle mass and strength underpins the development of therapies to prevent or offset sarcopenia and associated co-morbidities. Muscle mass and strength also are determining factors of athletic performance^2^. Despite females representing approximately 50% of the human population, research in the field of skeletal muscle regulation and the response to exercise has been overwhelmingly performed on male cohorts. Between 2017 and 2019, only 8% of all sports and exercise research was made up of female-only cohorts and the majority of these tend to relate to aspects specific to females, such as pregnancy, menopause or reproductive disease^3^. However, male and female muscle physiology differ in many ways. For example, the growth and regenerative capacity of skeletal muscle vary between males and females^4^. Male myocytes exhibit greater proliferative capacity, while female myocytes display greater differentiation in vitro^4^. There are also sex-specific differences in skeletal muscle morphology, where females have more type I muscle fibres, while males have more type IIb muscle fibres^5^. In response to resistance training, females display greater fatigue resistance and a greater capacity for neural adaptations when compared to males^6^. These differences are driven, in part, by varying concentrations of the major sex hormones, oestrogen and testosterone^4,5^.

The major androgen hormone testosterone is an anabolic hormone that regulates skeletal muscle growth. Testosterone exerts its effects on target tissues, including skeletal muscle, by binding to its specific receptor, the androgen receptor (AR)^7^. Testosterone is also present in females, albeit at concentrations about tenfold lower than typical male levels^8^. In pre-menopausal females, testosterone is mostly active in the regulation of the reproductive and nervous systems^8^; however, its role in the regulation of skeletal muscle growth is not well understood. Despite having about tenfold less testosterone than males of a similar age, pre-menopausal females exhibit similar relative strength^9^ and muscle mass gains^10^ as their male counterparts in response to resistance training. Protein synthesis and degradation rates are also similar between males and females, both at rest and after resistance exercise^11^. There is further evidence from mouse studies to suggest that testosterone and other androgen hormones may not be necessary to reach peak muscle mass or strength in females^12^. Instead, growth hormone (GH), insulin-like growth factor-1 (IGF-1) and oestrogen may take over some of the anabolic role of testosterone in females^12,13^.

In untrained young males, a moderate-to-strong positive relationship exists between testosterone concentrations, lean body mass and muscle strength, when expressed relative to body mass^14,15^. In young healthy men (n = 61), testosterone concentrations correlated with fat-free mass, leg muscle size and strength in a dose-dependent manner when testosterone concentrations were pharmaceutically manipulated for 20 weeks^15^. This holds true for endogenous testosterone, where men with high testosterone concentrations have more relative lean mass than those with low testosterone concentrations (n = 252)^14^. Limited evidence about the relationship between testosterone, muscle mass and muscle strength is currently available in pre-menopausal females. Administration of exogenous testosterone that raised testosterone levels by approximately four-fold for 10 weeks resulted in increased lean mass and running time to exhaustion^16^, but did not alter body fat percentage, VO_2max_ or functional outcomes including leg muscle strength and power and anaerobic power^16^. The relationship between endogenous testosterone and muscle-related outcomes has not been investigated using large cohorts of healthy, pre-menopausal females using appropriately adjusted models.

The aim of this study was to examine cross-sectional evidence of relationships between endogenous testosterone concentrations, lean mass and handgrip strength in 18–40-year-old premenopausal females from the National Health and Nutrition Examination Survey (NHANES). It was hypothesised that there would be no associations between total testosterone and lean mass or handgrip strength in this population. A secondary hypothesis was that free androgen index (FAI), reflective of the amount of ‘free’ testosterone, would be associated with lean mass and handgrip strength in pre-menopausal females.

## Methods

### Study population

NHANES is a nationally representative, cross-sectional survey conducted in the United States of America that has run annually since 1971. NHANES uses a multi-stage, stratified, clustered probability sample including non-institutionalised civilians over two months of age. Further information about sampling, study design and all protocols can be found at https://www.cdc.gov/nchs/nhanes. Briefly, NHANES consists of an initial at-home interview, where trained staff ask questions with automated data collection^17^. All participants then attend a mobile examination clinic (MEC) where trained staff collect anthropometric data and biological samples^18^. This study used the cohort recruited in 2013–2014, where 10,175 individuals participated in the at-home interviews. Of these individuals, 9,813 participated in the MEC (96%).

Individuals were excluded from the cohort if they were male (n = 5003), and if they were younger than 18 (n = 1975) or older than 40 (n = 1949) years of age. This age range includes young to middle-aged females that were not menopausal, as menopause may affect the relationship between testosterone and skeletal muscle due to the significant decrease in oestrogen and sex hormone binding globulin (SHBG) that occurs at this time^19^. Females who were pregnant (n = 63) or who had not had regular menses in the last 12 months due to menopause (n = 1) were excluded. Females with previous diagnoses of cancer (n = 34), thyroid conditions (n = 69) or chronic obstructive pulmonary disease (n = 3) were also excluded due to the long-term effects of these conditions on skeletal muscle mass^20–22^. The decision to exclude individuals who reported taking anabolic steroids was made a priori, however, no individuals in this dataset met this criterion. The final female cohort consisted of 716 pre-menopausal females aged 18–40 years. A sensitivity analysis comparing the results of females who have previously used exogenous female hormones and those who have not was conducted (supplementary Tables 1–4). The results of these sub-cohorts were broadly consistent with the results from the entire cohort. For this reason, only the results from the entire cohort will be discussed below.

**Table 1:** Weighted characteristics of included females.

Variable	Mean ± SD (n = 716 females)	Range (min–max)
Age (years)	29.6 ± 6.4	18–40
Ethnicity (%)		
Non-hispanic white	35.9	
Non-hispanic black	21.7	
Non-hispanic asian	12.8	
Other non-hispanic	4.8	
Mexican hispanic	15.3	
Other hispanic	9.4	
BMI (kg m^2^)	28.5 ± 7.7	16.1–60.9
Lean Index (LMI; kg·m^2^)	16.4 ± 3.0	10.6–30.5
Height-adjusted upper body lean mass index (UBLMI; kg·m^2^)	1.7 ± 0.4	1.0–3.3
Height-adjusted lower body lean mass index (LBLMI; kg·m^2^)	5.2 ± 1.1	3.0–11.4
Fat percentage (%)	37.7 ± 6.1	18.8–52.8
[Testosterone] (nmol·L^−1^)	1.0 ± 0.6	0.1–5.3
[Oestrogen] (pg·mL^−1^)	94.0 ± 79.3	9.0–513.0
[SHBG] (nmol·L^−1^)	81.4 ± 62.0	8.9–452.3
Free Androgen Index (FAI)	1.90 ± 2.11	0.1–23.5
Combined handgrip strength (kg)	61.7 ± 10.5	22.6–99.7
Protein intake (g·day^−1^)	73.0 ± 34.0	3.0–296.2
Total vitamin C intake (mg·day^−1^)	72.0 ± 71.4	0.0–796.3
Total vitamin D intake (mcg·day^−1^)	4.0 ± 5.4	0.0–62.4
Total magnesium intake (mg·day^−1^)	262.1 ± 137.5	36–2725
Female hormone use (%)		
No	32.0	
Yes	68.0	
Average total physical activity (MET-min·week^−1^)	3 347 ± 5 636	0–45,600
Time of venepuncture (%)		
Morning (fasted)	45.3	
Afternoon	29.8	
Evening	25.0	
Alcohol consumption (%)		
< 12 drinks in life	9.6	
≥ 1 drink on 1–3 days·month^−1^	39.6	
≥ 1 drink on1-3 days·week^−1^	40.2	
≥ 1 drink on 4 + days·week^−1^	10.6	

Values are mean ± standard deviation. n = 716.

### Ethical approval and consent procedures

The National Centre for Health Statistics (NCHS) Ethics Review Board (ERB) found all NHANES protocols to comply with the U.S. Health and Human Services policy for Protection of Human Research Subjects and all NHANES protocols were approved (protocol number #2011-17) and found to comply with all relevant regulations. NHANES is comprised of two phases and signed, informed consent was sought from every participant before each stage^23^.

The Deakin University Human Research Ethics Committee (DUHREC) determined that this study met the National Statement on Ethical Conduct in Human Research criteria for negligible risk research and therefore exempted the secondary data analysis undertaken for this study from further ethics review.

### Procedures and measures

#### Demographic and health information and behaviours

NHANES interviewers collected information about age, race, gender, medical history, reproductive health, dietary information, alcohol consumption and physical activity levels about all the individuals in the household. This information was gained through standardised questionnaires delivered by a trained interviewer, according to NHANES protocol^17^, which can be found at https://www.cdc.gov/nchs/nhanes. Physical activity data was collected using the Global Physical Activity Questionnaire (GPAQ). Dietary information was collected via a 24-h recall administered by NHANES interviewers. The alcohol use variable was synthesised from answers to questions from the home interviews. Individuals were categorised into: < 12 drinks in lifetime (very infrequent), having at least one drink on one to three days per month (infrequent), having at least one drink on one to three days per week (moderate), or having at least one drink on four to seven days per week (frequent). Participants then attended the MEC for a physical examination.

#### Hormone analysis

Before arriving at the MEC, participants were randomly assigned to morning, afternoon or evening sessions. Participants in the morning sessions fasted for at least nine hours; those attending afternoon or evening sessions had no dietary restrictions^17^. A trained phlebotomist collected blood according to relevant regulations and NHANES protocol^18^. Testosterone, oestrogen and SHBG were assessed via isotope dilution liquid chromatography tandem mass spectrometry (ID-LC-MS/MS)^22^. Insulin measures were taken in participants who took part in the morning session of the MEC and were fasting. Insulin was measured using a two-site immunoenzymometric assay. The lower limit of detection (LLOD) for testosterone, oestrogen, SHBG and insulin analyses were 0.026 nmol·L^−1^, 10.987 pmol·L^−1^, 0.8 nmol·L^−1^ and 6 pmol·L^-1^, respectively^24^. The lower limit of quantification (LLOQ) was estimated by multiplying the LLOD by three.

#### Body composition analysis

Body composition was assessed via dual-energy x-ray absorptiometry (DXA) during the MEC^18^. Scans were acquired with the Hologic Discovery model A densitometers (Hologic, Inc., Bedford, Massachusetts), using software version Apex 3.2. Individuals were not eligible for a DXA scan if their weight or height exceeded 450 lb (204.11 kg) or 6′5″ (195.58 cm), respectively^18^. In-depth protocols for DXA scans are located at https://www.cdc.gov/nchs/nhanes.

#### Handgrip strength

The procedures to measure handgrip strength is described in detail at https://www.cdc.gov/nchs/nhanes. Briefly, participants squeezed a dynamometer as hard as possible with their dominant hand, in a standing position^18^. The test was repeated on the other hand, and then twice more for each hand. Exactly sixty seconds separated attempts on the same hand. Combined grip strength was the sum of the largest reading from each hand^18^ and was used in our final analyses.

### Data cleaning and manipulation

Testosterone concentrations were converted to SI units (nmol·L^−1^) by dividing all data by 28.818. Any hormone data that were below the LLOQ of the ID-LC-MS/MS were removed. No individuals had testosterone readings below the LLOQ, 76 individuals had oestrogen levels below the LLOQ and no individuals had SHBG levels below the LLOQ. Implausible hormone values (pragmatically defined as values that were > mean ± eight × SD) were also coded missing. One female had testosterone levels over 19 nmol·L^−1^, one female had a SHBG value of over 1000 nmol·L^−1^, and three females had oestrogen readings over 700 pg·mL^−1^; all of these values were accordingly coded as missing.

Total physical activity levels were calculated according to NHANES and Global Physical Activity Questionnaire (GPAQ) guidelines^17^. The average minutes per week was calculated for each discrete physical activity domain (vigorous or moderate work, transport and vigorous or moderate leisure time) and converted to metabolic equivalents (METS). Vigorous activity was classified at eight METS and moderate or transport physical activity was classified as four METS, as per NHANES protocol^17^. The domain-specific MET scores were then summed to generate a total physical activity measurement in MET-minutes per week. Information regarding muscle-strengthening activities was not recorded in this cohort and therefore was not included in our analyses.

Height-adjusted lean mass, or lean mass index (LMI; kg·m^2^), was calculated by dividing total body lean mass (excluding bone mineral content) in kg by height in metres, squared. Lean mass (%) was calculated by dividing total body lean mass (excluding bone mineral content) by body mass and multiplying by 100. Upper body lean mass index (UBLMI; kg·m^2^) was calculated by summing the lean mass for the right and left arms and dividing by height in metres, squared. This measurement was used to measure the appendicular lean mass of the upper body only. This excludes organ mass, which may be influenced factors including hydration status, and provides a more accurate depiction of muscle mass of the upper body^24^. Lower body lean mass index (LBLMI; kg·m^2^) was calculated by summing the lean mass for the right and left legs and dividing by height in metres, squared. Free androgen index (FAI) was calculated by dividing total testosterone (nmol·L^−1^) by SHBG (nmol·L^−1^) and multiplying by 100.

All independent (total testosterone, FAI, SHBG) and dependent (LMI, UBLMI, LBLMI, handgrip strength) variables were standardised by calculating the z-score for each variable (mean = 0, SD = 1). Standardised variables were used for subsequent analyses, allowing for estimation of the magnitude of any significant relationship.

### Statistical analyses

All statistical analyses were performed with Stata software version 15.0 (StataCorp, College Station, TX) and accounted for the complex survey design and stratification employed by NHANES by using the appropriate sample design variables (strata and primary sampling unit). The one-day dietary weighting scheme was applied to account for oversampling of different populations and yield estimates representative of the US population, according to NHANES data analysis guidelines, found at https://www.cdc.gov/nchs/nhanes. This scheme was chosen as it relates to the smallest sampling unit, as per NHANES protocol. For a sensitivity analysis using participants with insulin data available, the fasting sampling weight was used, as per NHANES guidelines.

Missing data were examined, and no patterns of missing data were identified. Under a missing at random assumption, multiple imputation by chained equations with predictive mean matching (using five nearest neighbours) was used to handle the missing data. Thirty imputations were used, based on 30% of participants having at least one missing data point for the study variables. All the analysis variables were used in the imputation model, with no additional auxiliary variables.

Multiple linear regression was performed to examine the relationship between testosterone, FAI and SHBG (independent variables) and handgrip muscle strength and lean mass (dependent variables) separately. Initial models included both linear and quadratic terms for testosterone, FAI, or SHBG, to account for potential curvilinear relationships. If there was insufficient evidence of a quadratic effect (*p*-values > 0.1), a linear model (with no quadratic effect) was tested.

Covariates included in the analysis were: total physical activity, ethnicity, historical female hormone use, oestrogen, SHBG, age, body fat percentage, alcohol consumption and the examination session to which each participant was assigned (morning, afternoon or evening; this also takes into account fasting status, as only the morning group were fasted). The session in which participants were examined was added as a covariate to control for the diurnal variation and the effects of fasting on testosterone concentrations and handgrip strength^25^. Historic exogenous female hormone administration (for contraception or otherwise) was added as a covariate to control for the effects of hormonal treatments on both sex steroid concentrations and lean mass. Dietary protein, vitamin C and D and magnesium intake were also accounted for as previous studies have shown relationships between lean mass and dietary protein, vitamin C and D and magnesium intake in females^26–29^. Height was added as a covariate in models assessing the effect of hormones on handgrip strength.

Collinearity was assessed by variance inflation factors, with a threshold of three set. No variables suggested inappropriate collinearity. All data are represented as mean ± SD. Statistical significance was set at *p* < 0.05. All exposure and outcome variables were standardised to assess the effect size of any significant relationships. Coefficients < 0.2 were considered ‘small’ effects, 0.2 < 0.5 were considered ‘medium’ effects, and 0.5 < 0.8 were considered ‘large’ effects^30^.

## Results

Our final cohort consisted of 716 females aged 18–40 years, with a mean age of 30 years. Mean testosterone concentration was 1.0 nmol·L^−1^ (range 0.1 to 5.3), mean LMI was 16.4 kg·m^−2^ (range 10.6 to 30.5) and mean combined handgrip strength was 61.7 kg (range 22.6 to 99.7). Over two-thirds (68.6%) of participants had taken female hormones throughout their life, either for contraception or other uses. The full characteristics of the study population are shown in Table 1.

In our cohort, there was no evidence of quadratic effects of total testosterone on total LMI, UBLMI, LBLMI or handgrip strength (all *p-*values > 0.1). There were also no significant linear effects of total testosterone on LMI, UBLMI, LBLMI or handgrip strength in either adjusted or unadjusted models. These results are shown in Table 2.

**Table 2:** Standardised linear effect of total testosterone on lean index (LMI), upper body lean mass index (UBLMI), lower body lean mass index (LBLMI) or combined handgrip strength in 18–40-year-old females (n = 716).

Variable (linear term)	Unadjusted linear model		Adjusted linear model	
	β (95% CI)	*p*	β (95% CI)	*p*
LMI	0.07 (− 0.04, 0.17)	*0.189*	0.05 (− 0.04, 0.15)	*0.237*
UBLMI	0.09 (0.00, 0.18)	*0.061*	0.06 (− 0.03, 0.16)	*0.171*
LBLMI	0.10 (− 0.01, 0.21)	*0.083*	0.05 (− 0.04, 0.15)	*0.228*
Combined handgrip strength	0.06 (− 0.03, 0.15)	*0.162*	0.01 (− 0.11, 0.12)	*0.926*

Conversely, there was a quadratic effect of FAI, a measure of testosterone that is not bound to SHBG and may be therefore considered the ‘free’ portion of testosterone, on LMI (β = − 0.03; *p* = 0.000), UBLMI (β = − 0.04; *p* = 0.000) and LBLMI (β = − 0.02; *p* = 0.001) in the models adjusted for all appropriate covariates (historical administration of exogenous female hormones, weekly physical activity habits, age, dietary protein, vitamin C, vitamin D and magnesium intake, oestrogen and SHBG concentrations, ethnicity, fat percentage, time of testing session and habitual alcohol consumption). These quadratic relationships suggest that LMI, UBLMI and LBLMI increase with increasing FAI. The relationship is steepest at lower FAI levels and the curve flattens with increasing FAI levels. There was no evidence of linear or quadratic effects of FAI on handgrip strength in the adjusted model. The linear effects of FAI on LMI, UBLMI, LBLMI and handgrip strength in both unadjusted and adjusted models are presented in Table 3.

**Table 3:** Standardised linear effect of free androgen index (FAI) on lean mass index (LMI), upper body lean mass index (UBLMI), lower body lean mass index (LBLMI) or combined handgrip strength in 18–40-year-old females (n = 716).

Variable	Unadjusted linear model		Adjusted linear model	
	β (95% CI)	*p*	β (95% CI)	*p*
**LMI**				
Quadratic term	–	***–***	− 0.03 (− 0.05, − 0.02)	0.000*
Linear term	0.24 (0.03, 0.45)	0.031*	0.02 (− 0.08, 0.25)	*0.276*
**UBLMI**				
Quadratic term	–	***–***	− 0.04 (− 0.05, − 0.02)	0.000*
Linear term	0.24 (0.03, 0.45)	0.029*	0.09 (− 0.08, 0.26)	*0.278*
**LBLMI**				
Quadratic term	–	***–***	− 0.02 (− 0.04, − 0.01)	0.001*
Linear term	0.23 (0.02, 0.44)	0.033*	0.08 (− 0.07, 0.22)	*0.260*
**Combined handgrip strength**	0.16 (0.08, 0.24)	0.001*	0.06 (− 0.02, 0.15)	*0.137*

*denotes statistical significance (*p* < 0.05).

Although it was originally included only as a covariate and not a primary outcome of this study, the observed positive relationships between FAI and LMI, UBLMI and LBLMI led us to conduct further analyses on SHBG. In females, SHBG was negatively linearly associated with LMI (β = − 0.13; *p* = 0.003), UBLMI (β = − 0.15; *p* = 0.002) and LBLMI (β = − 0.09; *p* = 0.018) after models were adjusted for all appropriate covariates (Table 4). SHBG was not associated with combined handgrip strength in an adjusted linear or quadratic model.

**Table 4:** Standardised linear effects of sex hormone binding globulin (SHBG) on lean mass index (LMI), upper body lean mass index (UBLMI), lower body lean mass index (LBLMI) or combined handgrip strength in 18–40-year-old females (n = 716).

Variable	Unadjusted linear model		Adjusted linear model	
	β (95% CI)	*p*	β (95% CI)	*p*
**LMI**				
Quadratic term	–	***–***	0.03 (0.00,0.07)	*0.065*
Linear term	− 0.25 (− 0.33, − 0.18)	0.000*	− 0.13 (− 0.21, − 0.06)	0.003*
**UBLMI**				
Quadratic term	–	***–***	0.04 (0.00,0.08)	0.038*
Linear term	− 0.25 (− 0.33, − 0.18)	0.000*	− 0.15 (− 0.22, − 0.67)	0.002*
**LBLMI**	− 0.21 (− 0.28, − 0.14)	0.000*	− 0.09 (− 0.16, − 0.02)	0.018*
**Combined handgrip strength**	− 0.10 (− 0.17, − 0.03)	0.009*	− 0.03 (− 0.10, 0.03)	*0.270*

*denotes statistical significance (*p* < 0.05).

Oestrogen may reduce muscle protein breakdown and increase muscle sensitivity to anabolic stimuli in females^13^. We therefore explored the possibility of a relationship between oestrogen and LMI, UBLMI, LBLMI and handgrip strength. However, there was no evidence for linear of quadratic effects of oestrogen on LMI, UBLMI, LBLMI or handgrip strength (all *p-*values > 0.1).

Previous literature suggests that insulin may mediate the relationship between testosterone and muscle-related outcomes in females^31^. For this reason, a sensitivity analysis was conducted on a smaller sub-cohort of females that were tested in the morning session of the MEC and from whom insulin measures were taken (n = 150). In this cohort, insulin was added as a covariate to statistical modelling. When insulin was added as a covariate, total testosterone was not related to any outcome in this sub-cohort (supplementary Table 5). FAI was not related to LMI, UBLMI or LBLMI in either linear or quadratic models but was associated with handgrip strength (β = 0.22; *p* = 0.001), which differs from the larger cohort (supplementary Table 6). Finally, SHBG was not associated with LBLMI, which differs from what was observed in the larger cohort (supplementary Table 7).

## Discussion

Despite the essential role of skeletal muscle for whole-body movement and metabolism, our understanding of the role of testosterone in muscle mass and strength has been mostly gained from male-only cohorts, warranting female-specific investigations. In line with our hypothesis, data from pre-menopausal females (aged 18–40 years) of the NHANES dataset indicate that total testosterone is not associated with LMI or handgrip strength in females. To our knowledge, this relationship has not been tested in a large, representative population of healthy, pre-menopausal females before.

Previous, smaller studies have suggested that total testosterone is not related to lean mass in young females. Total testosterone was not associated with lean mass in healthy 18–40-year-old females (n = 185)^31^. Furthermore, in lean females (BMI approximately 22 kg·m^−2^) aged 17–21 years with ovarian hyperandrogenism, defined by amenorrhea or oligomenorrhea and/or hirsutism, lean mass was significantly reduced when compared to healthy controls (n = 22 per group)^32^. This finding was replicated in a small cohort of lean (BMI < 25 kg·m^−2^) females aged 18–30 suffering from polycystic ovary syndrome (PCOS; n = 10 per group)^33^. PCOS is the most common cause of hyperandrogenism in females and affects as much as 4–10% of reproductive-aged females^34^ and 20–37% of elite female athletes^35^. PCOS participants had significantly lower lean mass than their weight and BMI-matched, healthy counterparts, despite having higher testosterone concentrations and similar levels of SHBG, oestradiol, follicle-stimulating hormone and luteinising hormone (n = 10 per group)^33^. In contrast, a small study observed a positive association between LMI and total testosterone in young females with PCOS (n = 48)^36^, where only subjects with a LMI above 14 kg·m^−2^ displayed significantly higher total testosterone^36^. Of importance, this model was not adjusted for potential confounders such as SHBG, physical activity or diet, all of which may affect lean mass^26–29,31^.

In vivo, testosterone exists either ‘free’ and unbound, or bound to proteins, such as albumin or SHBG^37^. It was historically assumed that only the ‘free’ form of testosterone was able to exert its effects on cells. There is however evidence suggesting that protein-bound steroids can also activate anabolic pathways, such as the Akt/mTOR pathway in rat myocytes in vitro^38^. Protein-bound sex steroids can also be internalised by cells via endocyctosis in female and male mice, suggesting that they may be biologically active^39^. In population studies, the free fraction of androgens is more commonly related to muscle mass and strength in males and females than the total concentration^40^. In line with the existing literature, our results provided evidence for a positive association between FAI, defined as the ratio between total testosterone and SHBG levels, and LMI, UBLMI and LBLMI. FAI has been previously positively associated with lean mass in 18–40 year old females (n = 95 PCOS patients, 90 healthy controls)^31^; a relationship that dissipated when the model was adjusted for insulin^31^. Insulin may mediate SHBG levels by decreasing hepatic SHBG production^41^, thereby influencing the FAI and its association with lean mass in females. Females suffering from PCOS with high insulin and IGF-1, another anabolic hormone, consistently display low SHBG concentrations^42–45^.

One limitation of our model is that it was not adjusted for growth hormone (GH), insulin or IGF-1, as insulin was only measured in females who took part in the morning session of the MEC (n = 150 with complete insulin data), while IGF-1 and GH were not measured by NHANES at all. However, sub-group analyses using insulin data in females from the morning session of the MEC suggest that the relationship between FAI and LMI, UBLMI and LBLMI dissipates when insulin is added as a covariate. This is in line with previous results^31^, suggesting that insulin is a moderator of this relationship. The relationship between SHBG and LBLMI also dissipated when insulin was added as a covariate, possibly because of the mediating role of insulin on SHBG and FAI levels^41^. Interestingly, a positive relationship between FAI and handgrip strength appeared in this sub-cohort when adjusted for insulin. While this may result from a statistical bias due to the considerably smaller cohort size (n = 150), it is important to note that females that were tested for insulin levels were fasted and were all tested in the morning session. Upon deeper analysis, the time of testing session (morning, afternoon or evening) was a significant moderator of the relationship between FAI and handgrip strength (*p* < 0.01) and may explain the different results to the larger cohort, where females were tested at all times of day. A reason for this may be the diurnal fluctuations of testosterone concentrations which, in females, can decrease by 25% across the day^46^. Alternatively, these results may simply show that insulin is a confounder of this relationship.

In line with these results, we also found evidence of a negative relationship between SHBG and LMI, UBLMI and LBLMI. Taken together, our results and others^31^ suggest that, in young females either healthy or suffering from PCOS (n = 95), the regulation of lean mass in pre-menopausal females may be more strongly mediated by SHBG, via its capacity to bind to testosterone, than by total testosterone itself. Evidence however suggests that SHBG may be more than simply a transport protein^39,47^. Indeed, a SHBG receptor exists on the membrane of rat skeletal muscle^47^. Upon ligand binding, this receptor activates cAMP as a secondary messenger to regulate the actions of androgens on their target cells^47,48^. SHBG might therefore also mediate the actions of steroids in vivo and provides interesting opportunities for future research*.* While FAI and SHBG were associated with LMI and UBLMI, the small coefficients (β-values < 0.2) should however be kept in mind as they suggest that only a minor proportion of the variability of LMI, UBLMI and LBLMI can be explained by steroid concentrations. This highlights the complexity of human physiology, where a myriad of different internal and external factors influence muscle health.

In line with our hypothesis, there was no evidence of relationships between handgrip strength and total testosterone, SHBG or FAI. This is an important set of findings, as the functional capacity of a muscle, measured by muscle strength, is arguably more important than muscle size in both athletic and every-day circumstances. Females can exhibit significant increases in muscular strength with training without a concurrent increase in muscle mass^6^. This suggests that neural adaptations are primary drivers of strength gains in females, rather than muscle hypertrophy^6^. In young females, muscle strength may therefore be a more important determinant of athletic performance than muscle mass, as previously shown in older adults^49^. Our results indicating no associations between markers of androgenicity and handgrip strength in females indicate that, while FAI and SHBG are related to LMI, this may not necessarily translate to muscle strength, specifically handgrip strength. It should however be noted that, despite being a commonly used and robust measure of overall muscular strength^50^, handgrip strength is not a perfect proxy for whole-body muscle strength^51^ and this should be accounted for when interpreting the results.

Recently, endogenous testosterone levels have been used as an eligibility criterion for specific athletic events in female sports^52^. Hyperandrogenic athletes (defined as females with testosterone levels over five nmol·L^−1^^52^) are banned from competing in specific athletic running events on the bases of their naturally-occurring total testosterone levels and the assumption of a direct association with athletic performance^52^. Our data suggest that total testosterone is not related to muscle mass or strength in females. Taken together, our data and others^31–33^ suggest that more evidence is needed to validate such relationships in both females with typical androgen levels and hyperandrogenic females.

When compared to the previous literature, the current study includes an unprecedentedly large sample size that is representative of the American population. Furthermore, the models used in this study have been adjusted for a number of covariates that can influence lean mass in females, which constitutes a strength of our analyses when compared to previous research. Using LMI and FAI in our models also provides a more physiologically relevant picture of the relationship between androgens, muscle mass and muscle strength in females. LMI accounts for the height of individuals, as opposed to lean mass as an absolute measure.

Limitations of this study include the cross-sectional, observational nature of the data, which prevents inferring causal relationships. In addition, NHANES does not include GH or IGF-1 measures. These anabolic hormones may play a role in the regulation of lean mass or muscle strength in females and should be accounted for as covariates when possible. Finally, NHANES does not include specific information about muscle strengthening activities in adults and therefore this could not be accounted for in the statistical modelling. However, total physical activity, which takes into account moderate and vigorous transport, work and leisure physical activity was added as a covariate. Another limitation of this study is that our strict inclusion and exclusion criteria may limit the direct generalization of our results to the wider population of females. In conclusion, our data indicate that total testosterone is not related to LMI, UBLMI, LBLMI or handgrip strength in pre-menopausal females, suggesting that testosterone is not a direct determinant of lean mass or muscle strength in this population. Our findings also indicate a positive relationship between FAI and lean mass, and a negative relationship between SHBG and lean mass. When compared to the total pool, ‘free’ testosterone concentrations may therefore be more highly associated with lean mass in females. Further, longitudinal or interventional research is warranted to better understand these relationships.

## Supplementary Information

Supplementary Information 1.

Supplementary Information 2.

Supplementary Information 3.

Supplementary Information 4.

Supplementary Information 5.

Supplementary Information 6.

Supplementary Information 7.

Supplementary Information 8.

## Data Availability

This study utilised freely available data from the Centre for Disease Control and Prevention for the National Health and Nutrition Examination Survey, available at https://www.cdc.gov/nchs/nhanes/index.htm. Code written for statistical analysis is available upon request.
