# Supplementary material for: Total testosterone is not associated with lean mass or handgrip strength in pre-menopausal females
Source: Sci Rep. 2021 May 13;11:10226. doi: 10.1038/s41598-021-89232-1 (PMC8119405; doi:10.1038/s41598-021-89232-1)
Supplement: Supplementary file 1 — Supplementary Information 1. [file 41598_2021_89232_MOESM1_ESM.docx]

Supplementary Table 1. Weighted characteristics of included females, and comparison of included females with historic exogenous female hormone use and those without. Values are mean ± standard deviation. * denotes p<0.05 compared to no historical hormone use. n= 716 total, 247 no hormone use, 469 hormone use.

|  | All females | No hormone use | Hormone Use |
| --- | --- | --- | --- |
| Variable | Mean ± SD  (n=716 females) | Mean ± SD  (n=247 females) | Mean ± SD  (n=469 females) |
| Age (years) | 29.6 ± 6.4 | 28.3 ± 6.1 | 30.0 ± 6.5 * |
| Ethnicity (%)  Non-Hispanic White  Non-Hispanic Black  Non-Hispanic Asian  Other Non-Hispanic  Mexican Hispanic  Other Hispanic | 35.9  21.7  12.8  4.8  15.3  9.4 | 23.5  21.1  19.0  4.0  21.0  11.3 | 44.5  20.7  8.5  5.3  12.8  8.1 |
| BMI (kg·m^2^) | 28.5 ± 7.7 | 28.5 ± 7.9 | 28.3 ± 7.4 |
| Fat Free Mass Index (FFMI; kg·m^2^) | 16.4 ± 3.0 | 16.4 ± 3.2 | 16.4 ± 2.9 |
| Height-adjusted upper body lean mass (UBLM; kg·m^2^) | 1.7 ± 0.4 | 1.7 ± 0.4 | 1.7 ± 0.3 |
| Height-adjusted lower body lean mass (LBLM; kg·m^2^) | 5.2 ± 1.1 | 5.2 ± 1.2 | 5.2 ± 1.1 |
| Fat percentage (%) | 37.7 ± 6.1 | 37.9 ± 6.0 | 37.6 ± 6.2 |
| [Testosterone] (nmol·L^-1^) | 1.0 ± 0.6 | 1.0 ± 0.5 | 1.0 ± 0.57 |
| [Oestrogen] (pg·mL^-1^) | 94.0 ± 79.3 | 95.3 ± 81.3 | 92.1 ± 78.0 |
| [SHBG] (nmol·L^-1^) | 81.4 ± 62.0 | 60.4 ± 42.5 | 91.1 ± 67.8* |
| Free Androgen Index (FAI) | 1.90 ± 2.11 | 2.1 ± 1.6 | 1.8 ± 2.3 |
| Combined handgrip strength (kg) | 61.7 ± 10.5 | 62.4 ± 11.6 | 61.5 ± 9.9 |
| Protein intake (g·day^-1^) | 73.0 ± 34.0 | 68.5 ± 32.3 | 75.0 ± 34.4 |
| Total vitamin C intake (mg·day^-1^) | 72.0 ± 71.4 | 75.0 ± 74.8 | 70.7 ± 70.0 |
| Total vitamin D intake (mcg·day^-1^) | 4.0 ± 5.4 | 4.3 ± 5.6 | 3.8 ± 5.0 |
| Total magnesium intake (mg·day^-1^) | 262.1 ± 137.5 | 234.8 ± 100.7 | 273.3 ± 150.3* |
| Female hormone use (%)  No  Yes | 32.0  68.0 | 100  0 | 0  100 |
| Average total physical activity  (MET-min/week) | 3 347 ± 5 636 | 3 380 ± 4 763 | 3 475 ± 6 217 |
| Time of venepuncture (%)  Morning (fasted)  Afternoon  Evening | 45.3  29.8  25.0 | 37.0  35.14  27.84 | 48.5  26.9  24.6 |
| Alcohol Consumption (%)  <12 drinks in life  ≥1 drink on 1-3 days/month  ≥1 drink on 1-3 days/week  ≥1 drink on 4+ days/week | 9.6  39.6  40.2  10.6 | 12.4  42.6  34.7  10.3 | 8.6  38.7  41.8  10.7 |
