# Supplementary material for: Total testosterone is not associated with lean mass or handgrip strength in pre-menopausal females
Source: Sci Rep. 2021 May 13;11:10226. doi: 10.1038/s41598-021-89232-1 (PMC8119405; doi:10.1038/s41598-021-89232-1)
Supplement: Supplementary file 2 — Supplementary Information 2. [file 41598_2021_89232_MOESM2_ESM.docx]

Supplementary Table 2. Standardised linear effects of **total testosterone** on lean mass index (LMI), upper body lean mass index (UBLMI), lower body lean mass index (LBLMI) or combined handgrip strength in 18–40-year-old females who have **never used exogenous female hormones** (n=247). There was no quadratic effect of total testosterone on any variable.

| **Variable (linear term)** | **β (95% CI)** | ***p*** |
| --- | --- | --- |
| LMI | 0.07 (-0.07, 0.21) | *0.328* |
| UBLMI | 0.06 (-0.09, 0.21) | *0.407* |
| LBLMI | 0.05 (-0.09, 0.19) | *0.428* |
| Combined handgrip strength | 0.07 (-0.18, 0.32) | *0.567* |
