# Supplementary material for: Total testosterone is not associated with lean mass or handgrip strength in pre-menopausal females
Source: Sci Rep. 2021 May 13;11:10226. doi: 10.1038/s41598-021-89232-1 (PMC8119405; doi:10.1038/s41598-021-89232-1)
Supplement: Supplementary file 3 — Supplementary Information 3. [file 41598_2021_89232_MOESM3_ESM.docx]

Supplementary Table 3. Standardised linear effects of **free androgen index (FAI)** on lean mass index (LMI), upper body lean mass index (UBLMI), lower body lean mass index (LBLMI) or combined handgrip strength in 18–40-year-old females who have **never used exogenous female hormones** (n=247). There was no quadratic effect of free androgen index on any variable.

| **Variable (linear term)** | **β (95% CI)** | ***p*** |
| --- | --- | --- |
| LMI | 0.25 (0.09, 0.40) | ***0.004*** |
| UBLMI | 0.26 (0.11, 0.40) | ***0.003*** |
| LBLMI | 0.21 (0.08, 0.35) | ***0.005*** |
| Combined handgrip strength | 0.11 (-0.09, 0.31) | *0.265* |
