# Supplementary material for: Total testosterone is not associated with lean mass or handgrip strength in pre-menopausal females
Source: Sci Rep. 2021 May 13;11:10226. doi: 10.1038/s41598-021-89232-1 (PMC8119405; doi:10.1038/s41598-021-89232-1)

Supplementary Table 4. Standardised linear effects of **sex hormone binding globulin (SHBG)** on lean mass index (LMI), upper body lean mass index (UBLMI), lower body lean mass index (LBLMI) or combined handgrip strength in 18–40-year-old females who have **never used exogenous female hormones** (n=247).

| **Variable (linear term)** | **Β (95% CI)** | ***p*** |
| --- | --- | --- |
| LMI  Quadratic term:  Linear term: | 0.08 (0.01, 0.15)  -0.8 (-0.26, 0.10) | ***0.032***  *0.376* |
| UBLMI  Quadratic term:  Linear term: | 0.10 (0.03, 0.17)  -0.14 (-0.34, 0.06) | ***0.012***  *0.155* |
| LBLMI  Quadratic term  Linear term | 0.07 (0.00, 0.14)  -0.009 (-0.17, 0.15) | *0.063*  *0.905* |
| Combined handgrip strength  Quadratic term  Linear term | -0.21 (-0.48, 0.06)  -0.12 (-0.31, 0.08) | *0.111*  *0.205* |

Quadratic term for SHBG vs FFMI:


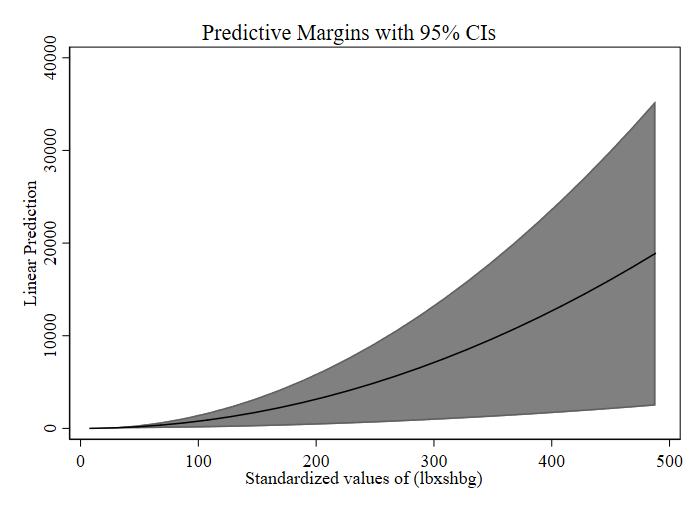

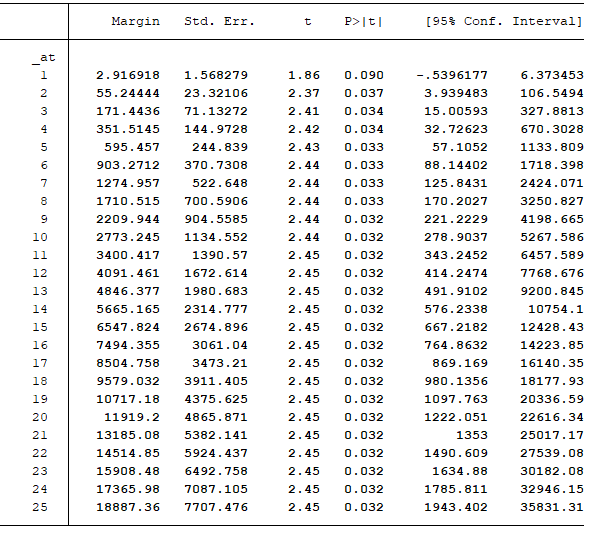


Quadratic term for SHBG vs UBLM


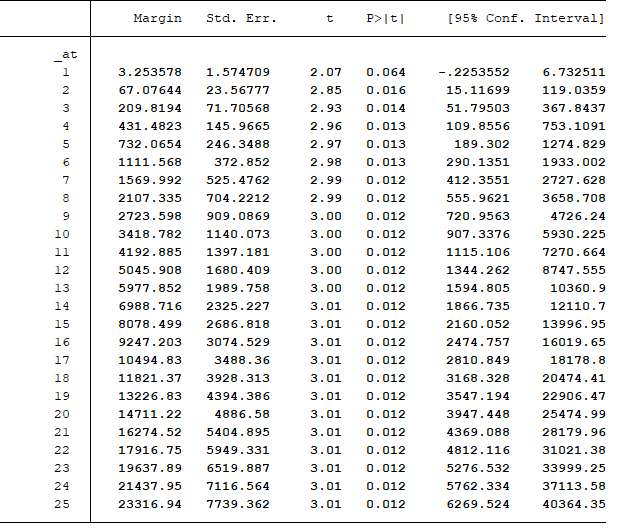


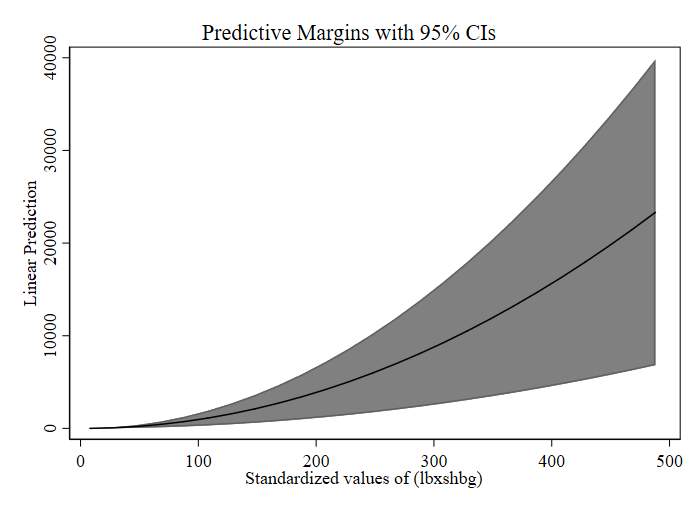

Supplement: Supplementary file 4 — Supplementary Information 4. [file 41598_2021_89232_MOESM4_ESM.docx]
