# Supplementary material for: Total testosterone is not associated with lean mass or handgrip strength in pre-menopausal females
Source: Sci Rep. 2021 May 13;11:10226. doi: 10.1038/s41598-021-89232-1 (PMC8119405; doi:10.1038/s41598-021-89232-1)
Supplement: Supplementary file 5 — Supplementary Information 5. [file 41598_2021_89232_MOESM5_ESM.docx]

Supplementary table 5. Standardised linear effect of **total testosterone** on lean index (LMI), upper body lean mass index (UBLMI), lower body lean mass index (LBLMI) or combined handgrip strength in 18-40 year old females with an added adjustment for insulin (n=150).

|  | **Adjusted linear model** | |
| --- | --- | --- |
| **Variable (linear term)** | **β (95% CI)** | ***p*** |
| LMI | 0.02 (-0.09, 0.14) | *0.671* |
| UBLMI | 0.02 (-0.06, 0.17) | *0.357* |
| LBLMI | 0.03 (-0.10, 0.16) | *0.625* |
| Combined handgrip strength | 0.02 (-0.17, 0.20) | *0.850* |
