# Supplementary material for: Total testosterone is not associated with lean mass or handgrip strength in pre-menopausal females
Source: Sci Rep. 2021 May 13;11:10226. doi: 10.1038/s41598-021-89232-1 (PMC8119405; doi:10.1038/s41598-021-89232-1)
Supplement: Supplementary file 6 — Supplementary Information 6. [file 41598_2021_89232_MOESM6_ESM.docx]

Supplementary table 6. Standardised linear effect of **free androgen index (FAI)** on lean mass index (LMI), upper body lean mass index (UBLMI), lower body lean mass index (LBLMI) or combined handgrip strength in 18-40 year old females with additional adjustment for insulin (n=150).

|  | **Adjusted model** | |
| --- | --- | --- |
| **Variable** | **β (95% CI)** | ***p*** |
| LMI  Quadratic term  Linear term | 0.00 (-0.06,0.05)  -0.07 (-0.21, 0.07) | *0.850*  *0.321* |
| UBLMI  Quadratic term  Linear term | -0.01 (-0.05, 0.04)  0.02 (-0.12, 0.15) | *0.753*  *0.812* |
| LBLMI  Quadratic term  Linear term | 0.01 (-0.07, 0.10)  -0.14 (-0.31, 0.02) | *0.671*  *0.081* |
| Combined handgrip strength | 0.22 (0.10, 0.33) | ***0.001*** |
