# Supplementary material for: Total testosterone is not associated with lean mass or handgrip strength in pre-menopausal females
Source: Sci Rep. 2021 May 13;11:10226. doi: 10.1038/s41598-021-89232-1 (PMC8119405; doi:10.1038/s41598-021-89232-1)
Supplement: Supplementary file 7 — Supplementary Information 7. [file 41598_2021_89232_MOESM7_ESM.docx]

Supplementary table 7. Standardised linear effects of **sex hormone binding globulin (SHBG)** on lean mass index (LMI), upper body lean mass index (UBLMI), lower body lean mass index (LBLMI) or combined handgrip strength in 18–40-year-old females with an additional adjustment for insulin (n=150).

|  | **Adjusted linear model** | |
| --- | --- | --- |
| **Variable** | **β (95% CI)** | ***p*** |
| LMI | -0.09 (-0.17, -0.01) | ***0.034*** |
| UBLMI | -0.10 (-0.19, -0.01) | ***0.028*** |
| LBLMI | -0.06 (-0.13, 0.02) | *0.134* |
| Combined handgrip strength | 0.02 (-0.08, 0.13) | *0.641* |
