# Supplementary material for: Total testosterone is not associated with lean mass or handgrip strength in pre-menopausal females
Source: Sci Rep. 2021 May 13;11:10226. doi: 10.1038/s41598-021-89232-1 (PMC8119405; doi:10.1038/s41598-021-89232-1)
Supplement: Supplementary file 8 — Supplementary Information 8. [file 41598_2021_89232_MOESM8_ESM.docx]

Table 1. Weighted characteristics of included females and wider NHANES cohort. Values are mean ± standard deviation.

|  | Cohort of interest | | Wider NHANES female cohort | |
| --- | --- | --- | --- | --- |
| Variable | Mean ± SD | Range  (min-max) | Mean ± SD | Range  (min-max) |
| Age (years) | 29.6 ± 6.4 | 18-40 | 32.1 ± 20.4 | 0 - 80 |
| Ethnicity (%)  Non-Hispanic White  Non-Hispanic Black  Non-Hispanic Asian  Other Non-Hispanic  Mexican Hispanic  Other Hispanic | 54.3  14.6  7.1  3.3  12.7  8.0 |  | 34.0  22.6  10.7  5.0  18.1  9.7 |  |
| BMI (kg·m^2^) | 28.5 ± 7.7 | 16.1 – 60.9 | 27.1 ± 8.6 | 12.1 - 82.9 |
| Lean Index (LMI; kg·m^2^) | 16.4 ± 3.0 | 10.6 – 30.5 | 16.0 ± 3.2 | 8.9 – 30.7 |
| Height-adjusted upper body lean mass index (UBLMI; kg·m^2^) | 1.7 ± 0.4 | 1.0 – 3.3 | 1.7 ± 0.4 | 0.8 – 3.4 |
| Height-adjusted lower body lean mass index (LBLMI; kg·m^2^) | 5.2 ± 1.1 | 3.0 – 11.4 | 5.1 ± 1.2 | 2.3 – 11.7 |
| Fat percentage (%) | 37.7 ± 6.1 | 18.8 – 52.8 | 37.4 ± 6.6 | 17.9 – 54.8 |
| [Testosterone] (nmol·L^-1^) | 1.0 ± 0.6 | 0.1 – 5.3 | 0.9 ± 0.8 | 0.1 – 9.5 |
| [Oestrogen] (pg·mL^-1^) | 94.0 ± 79.3 | 9.0 – 513.0 | 90.7 ± 87.9 | 9.04 – 615 |
| [SHBG] (nmol·L^-1^) | 81.4 ± 62.0 | 8.9 – 452.3 | 85.0 ± 77.0 | 5.18 – 758.8 |
| Free Androgen Index (FAI) | 1.90 ± 2.11 | 0.1 – 23.5 | 1.5 ± 1.6 | 0.0 – 34.2 |
| Combined handgrip strength (kg) | 61.7 ± 10.5 | 22.6 - 99.7 | 55.1 ± 14.32 | 8.0 – 102.4 |
| Protein intake (g·day^-1^) | 73.0 ± 34.0 | 3.0 - 296.2 | 66.6 ± 33.3 | 0.7 – 370.6 |
| Total vitamin C intake (mg·day^-1^) | 72.0 ± 71.4 | 0.0 - 796.3 | 72.6 ± 72.8 | 0.0 – 796.3 |
| Total vitamin D intake (mcg·day^-1^) | 4.0 ± 5.4 | 0.0 - 62.4 | 4.2 ± 4.7 | 0.0 – 62.4 |
| Total magnesium intake (mg·day^-1^) | 262.1 ± 137.5 | 36 - 2725 | 247.5 ± 127.9 | 10 – 2725 |
| Female hormone use (%)  No  Yes | 32.0  68.0 |  | 21.0  79.0 |  |
| Average total physical activity  (MET-min/week) | 3 347 ± 5 636 | 0 – 45 600 | 2 554 ± 4 444 | 0 – 45 600 |
| Time of venepuncture (%)  Morning (fasted)  Afternoon  Evening | 45.3  29.8  25.0 |  | 42.5  34.4  23.1 |  |
| Alcohol Consumption (%)  <12 drinks in life  ≥1 drink on 1-3 days/month  ≥1 drink on1-3 days/week  ≥1 drink on 4+ days/week | 9.6  39.6  40.2  10.6 |  | 20.2  36.2  32.0  11.6 |  |
